# Accumulation and Transport of 1-Aminocyclopropane-1-Carboxylic Acid (ACC) in Plants: Current Status, Considerations for Future Research and Agronomic Applications

**DOI:** 10.3389/fpls.2017.00038

**Published:** 2017-01-24

**Authors:** Lisa Vanderstraeten, Dominique Van Der Straeten

**Affiliations:** Laboratory of Functional Plant Biology, Department of Biology, Ghent UniversityGent, Belgium

**Keywords:** 1-aminocyclopropane-1-carboxylic acid, ACC, agriculture, conjugation, deaminase, ethylene, signal, transport

## Abstract

1-aminocyclopropane-1-carboxylic acid (ACC) is a non-protein amino acid acting as the direct precursor of ethylene, a plant hormone regulating a wide variety of vegetative and developmental processes. ACC is the central molecule of ethylene biosynthesis. The rate of ACC formation differs in response to developmental, hormonal and environmental cues. ACC can be conjugated to three derivatives, metabolized *in planta* or by rhizobacteria using ACC deaminase, and is transported throughout the plant over short and long distances, remotely leading to ethylene responses. This review highlights some recent advances related to ACC. These include the regulation of ACC synthesis, conjugation and deamination, evidence for a role of ACC as an ethylene-independent signal, short and long range ACC transport, and the identification of a first ACC transporter. Although unraveling the complex mechanism of ACC transport is in its infancy, new questions emerge together with the identification of a first transporter. In the light of the future quest for additional ACC transporters, this review presents perspectives of the novel findings and includes considerations for future research toward applications in agronomy.

## Acc, the Direct Precursor of the Plant Hormone Ethylene

1-aminocyclopropane-1-carboxylic acid (ACC) is a three-membered ring non-protein amino acid which is the direct precursor of the plant hormone ethylene. This gaseous plant hormone was identified as a regulator of plant growth in 1901 by Neljubov. Decades of dedicated research revealed a myriad of plant responses to ethylene (Abeles et al., 1992). This two-carbon atom molecule controls several processes linked to vegetative plant growth but is also a major player in seed germination, fruit ripening, leaf and flower senescence and abscission (Lin et al., 2009; Bakshi et al., 2015). During the above-mentioned processes ethylene production increases significantly in comparison to the relatively low basic levels. The regulation of seedling growth is one of the best characterized ethylene responses. First reported by Neljubov, and later confirmed by Knight and Crocker (1913), was the ethylene response of dark-grown seedlings, known as the triple response including (1) shortening of the hypocotyl and the root, (2) radial swelling of the hypocotyl, and (3) exaggeration of the apical hook.

**FIGURE 1:**
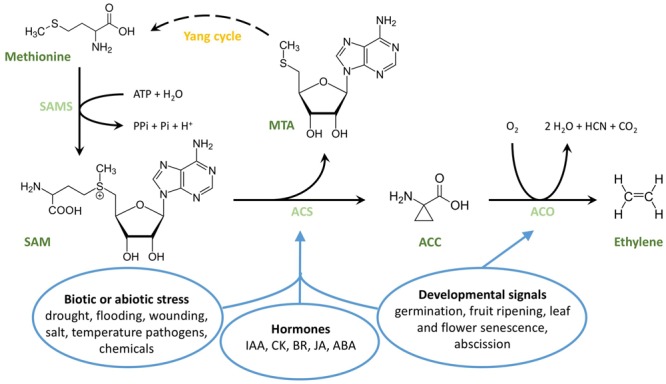
**Structural overview of ethylene biosynthesis.** The amino acid methionine is converted to *S*-adenosyl-L-methionine (SAM) by SAM-synthetase (SAMS), a reaction that requires ATP. SAM is then converted to ACC by ACC synthase (ACS), in a reaction that cleaves off a 5′methylthioadenosine (MTA). MTA is recycled back to methionine through a series of intermediate steps, known as the Yang cycle or Methionine Salvage Pathway. In the presence of oxygen, ACC is converted to ethylene by ACC oxidase (ACO). The ovals represent sets of endogenous and exogenous cues stimulating ethylene production. IAA, indol-3-acetic acid (Auxin); CK, cytokinin; BR, brassinosteroid; JA, jasmonic acid; ABA, abscisic acid.

Lieberman and Mapson (1964), Lieberman et al. (1965), Murr and Yang (1975), Adams and Yang (1977), and Adams and Yang (1979) made major contributions to our understanding of ethylene biosynthesis. A simplified overview of the pathway is presented in **Figure 1**. In higher plants, ethylene is produced from the amino acid methionine (Lieberman and Mapson, 1964; Lieberman et al., 1965), which is converted to *S*-adenosyl-L-methionine (SAM) (Burg, 1973) by SAM-synthetase (SAMS; also known as L-methionine-*S*-adenosyltransferase) (Murr and Yang, 1975; Adams and Yang, 1977, 1979). Subsequently, the three-membered ring amino acid 1-aminocyclopropane-1-carboxylic acid (Adams and Yang, 1979) is formed in a reaction catalyzed by the enzyme ACC synthase (ACS) (Boller et al., 1979). ACS is part of a family of PLP dependent enzymes, which require pyridoxal-5′-phosphate (PLP) as a cofactor. After binding of PLP to its catalytic site, ACS cleaves a 5′-methylthioadenosine (MTA) molecule from SAM, and induces the formation of the cyclopropane ring characteristic for ACC (Yu et al., 1979; Liang et al., 1992). MTA is recycled back to methionine through a set of different reactions known as the Methionine Salvage Pathway or Yang-cycle (Murr and Yang, 1975; Miyazaki and Yang, 1987; Burstenbinder et al., 2010). By recycling the sulfur and methyl group from SAM through the Yang-cycle, the plant is capable of producing high levels of ethylene without depleting the sulfur-containing methionine pool (Miyazaki and Yang, 1987). This process is essential as sulfur is a limiting compound for plants. In a final biosynthetic step, ACC is converted to ethylene in a reaction catalyzed by the enzyme ACC-oxidase (ACO), which was identified and characterized by John et al. (1985); Ververidis and John (1991).

## Control of the Acc Pool: Acc Accumulation, Acc Reduction, and Regulation Thereof

The levels of ethylene biosynthesis throughout the plant are relatively low during vegetative development, but are increased in response to a wide variety of developmental conditions as well as by several hormonal signals and environmental cues (**Figure 1**). Firstly, ethylene biosynthesis can be altered in response to developmental processes such as germination, fruit ripening, leaf and flower senescence, and abscission (Yang and Hoffman, 1984; Abeles et al., 1992). Secondly, it is regulated by plant hormones auxins, cytokinins, brassinosteroids, jasmonic acid, and abscisic acid (ABA) (Yang and Hoffman, 1984; Abeles et al., 1992; Vogel et al., 1998b; Woeste et al., 1999). Thirdly, ethylene can affect its own biosynthesis through positive and negative feedback loops. (Riov and Yang, 1982a,b; Kende, 1993; Nakatsuka et al., 1997, 1998). Finally, ethylene biosynthesis can be enhanced by biotic and abiotic stress signals such as flooding, wounding, drought, and pathogen attack (Morgan and Drew, 1997; Pierik et al., 2007; Kazan, 2015).

The majority of the regulatory mechanisms of ethylene biosynthesis act at the level of ACC production by ACS. However, there are additional regulatory mechanisms. Under conditions of high ethylene production, the pathway can also be regulated at the level of the conversion of ACC into ethylene by ACO. Conjugation and deamination of ACC regulates the pool of available ACC. In addition, the ACC pool is also indirectly altered by action of VAS1 (REVERSAL OF SAV3 PHENOTYPE 1). The *vas1* mutant, identified by Zheng et al. (2013) as a genetic suppressor of the auxin biosynthesis mutant *shade avoidance3* (*sav3*), links ethylene with auxin biosynthesis. VAS1 is an aminotransferase catalyzing the transamination of the auxin biosynthetic intermediate indole-3-pyruvic acid into the amino acid L-tryptophan. For this transamination reaction VAS1 uses the ethylene biosynthetic precursor methionine as an amino donor. VAS1 activity was linked to shade avoidance and it was proposed that the reduction in auxin and ethylene by VAS1 restricts elongation growth, providing a mechanism to prevent plants from over-reacting to shade.

### Regulation of the ACC Pool: ACC Production by ACS

The conversion of SAM to ACC by ACS is the major regulatory step in ethylene biosynthesis, hence all conditions which ultimately lead to ethylene formation cause an accumulation of ACC (Yang and Hoffman, 1984). As mentioned previously, ACS is a member of a superfamily of proteins requiring pyridoxal-5′-phosphate (PLP) as a cofactor, known as PLP-dependent proteins. In most plant species, ACS is encoded by a multigene family, the members of which show distinct but overlapping expression patterns. The *Arabidopsis* genome contains eight *ACS* genes encoding functionally active ACS enzymes (ACS2, ACS4-9, and ACS11), and a ninth catalytically inactive member, ACS1 (Liang et al., 1992; Van Der Straeten et al., 1992; ACS1 in the latter reference corresponds to ACS2 in the former). *ACS* mutant complementation by Tarun and Theologis (1998) suggested that the enzyme functions as a homodimer whose active site is formed through interaction of shared residues from the monomeric subunits. Capitani et al. (1999) confirmed this mode of action by study of the quaternary structure of an apple ACS protein. Besides functioning as homodimers, it was suggested that ACSs are also capable of forming heterodimers (Tarun and Theologis, 1998), which was corroborated by Tsuchisaka and Theologis (2004a). The latter study revealed that the ACS proteins can only form enzymatically active heterodimers among members of the same phylogenetic branch, while all functional ACS are homodimers with shared active sites. The capability of acting as homo- or hetero-dimers is a characteristic also present in other PLP-dependent enzymes.

In most plant species, the members of the *ACS* gene family are differentially regulated at the transcriptional level, in an organ-specific, tissue-specific and/or cell-type specific manner (Kende, 1993; De Paepe and Van Der Straeten, 2005). To date, most gene expression studies have been performed on an organ or tissue specific basis. Rodrigues-Pousada et al. (1993) performed a complete analysis of *ACS1* (termed *ACS2* in Liang et al., 1992) gene expression during *Arabidopsis* development using a GUS-reporter construct. Ishiki et al. (2000) investigated the *ACS* gene expression of etiolated melon seedlings (root, hypocotyl, and cotyledons) and melon fruit, Peng et al. (2005) investigated the *Arabidopsis ACS* gene expression in the root and the shoot, and Xue et al. (2008) determined *ACS* expression patterns in different rose floral tissues (sepals, petals, stamens, gynoecia, and receptacles). However less abundant, a number of examples can be found of cell type-specific gene expression analyses. Geisler-Lee et al. (2010) studied the *ACS* gene expression in different cell types in maize roots. Tsuchisaka and Theologis (2004b) looked both at the organ specific and cell-type specific *Arabidopsis ACS* expression patterns. In seedlings, similar but not identical expression patterns were found in the light and in darkness. Interestingly, no expression of *ACS9* is observed in both conditions, while in the light *ACS8* is the only gene expressed in the root tip. In mature plants, *ACS1* is mainly expressed in vascular tissue, *ACS2, 4, 5, 6*, and *8* are expressed in roots, inflorescence stem, siliques and younger leaves, *ACS11* is expressed in roots, inflorescence stem, younger leaves, cauline leaves, and *ACS9* is barely expressed. In their cell type-specific expression studies they analyzed tissues from cotyledons, the hypocotyl and the root. In cotyledons and the hypocotyl, expression of all genes, except *ACS1* and *ACS9*, is restricted to the epidermal cell layer, the vascular bundles and the guard cells. In the roots, three different zones were examined: lateral root cap, cell division, and cell expansion. *ACS8* is uniquely expressed in the lateral root cap zone. In the other two zones, most *ACS* expression is restricted to the endodermis, pericycle, and stele. However, IAA treatment enhanced the expression of *ACS7* and *ACS8* in the epidermis and of *ACS11* in all cell types. Using data from GeneAtlas and the AREXdb, these expression patterns were confirmed by Dugardeyn et al. (2008); however, they also revealed expression in the root cap for *ACS2, 4, 8, 10*, and *12*. This discrepancy with the analysis of Tsuchisaka and Theologis (2004b) probably results from limitations of the GUS reporter. Although little attention has been given to the exact mechanisms at the origin of these differences in expression levels, it is established that *ACS*s are differentially regulated in response to a variety of developmental or environmental signals and in response to plant hormones.

**FIGURE 2:**
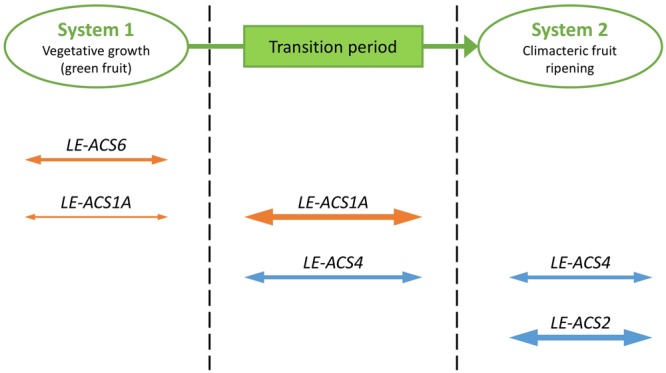
**Developmental regulation of *ACS* genes at the transcriptional level.** Cartoon representation of the expression of *ACS* genes during the transition from system 1 to system 2 ethylene biosynthesis in tomato fruit ripening. System 1 ethylene biosynthesis is regulated by the expression of *LE-ACS1A* and *LE-ACS6* in green fruit, followed by a transition period in which *LE-ACS1A* expression increases and *LE-ACS4* expression is induced. The elevated ethylene production results in a negative feedback on the system 1 pathway, reducing *LE-ACS1A* and *LE-ACS6* expression. System 2 ethylene biosynthesis is regulated by the expression of *LE-ACS2* and *LE-ACS4*. Relative levels of gene expression are presented by the size of the arrows; genes negatively regulated by ethylene are presented in orange; genes positively regulated by ethylene are presented in blue.

A first example illustrates how *ACS* genes in tomato are differentially regulated during fruit ripening. Similar to *Arabidopsis*, tomato *ACS* genes are part of a multigene family with differential expression patterns regulated by developmental, biotic and abiotic signals. When ripening starts in tomato or other climacteric fruits, the regulation of ethylene biosynthesis switches from auto-inhibitory to auto-stimulatory. In these plant species, two systems of ethylene production have been proposed (Barry et al., 2000; Alexander and Grierson, 2002). System 1 operates during vegetative growth, during which ethylene inhibits its own biosynthesis (auto-inhibition), while system 2 operates upon fruit ripening, during which ethylene induces its own biosynthesis (auto-catalysis). Barry et al. (2000) observed that the transcripts corresponding to four *ACS* genes, *LE-ACS1A, LE-ACS2, LE-ACS4*, and *LE-ACS6*, were detected in tomato fruit. A detailed expression analysis using the wild type and ripening mutants revealed that each of these *ACS* genes is regulated in a unique way. System 1 ethylene biosynthesis is regulated by the expression of *LE-ACS1A* and *LE-ACS6* in green fruit, followed by a transition period in which the MADS-Box Transcription Factor RIN (RIPENING INHIBITOR) plays an important role in increasing *LE-ACS1A* expression and inducing *LE-ACS4* expression (**Figure 2**). The increased ethylene production triggers the expression of *LE-ACS2*. Subsequently, autocatalytic system 2 is initiated. The elevated ethylene production results in a negative feedback on the system 1 pathway, reducing *LE-ACS1A* and *LE-ACS6* expression. System 2 is maintained by expression of *LE-ACS2* and *LE-ACS4* (Lincoln et al., 1993; Barry et al., 2000).

The next few examples illustrate that the expression of *ACS* genes show different susceptibilities to other plant hormones, and that these susceptibilities can differ between different cell types. Rodrigues-Pousada et al. (1999) provided the first evidence for transcriptional regulation of *ACS1* by auxin and cytokinin. Yamagami et al. (2003) demonstrated that indole-3-acetic acid (IAA) induces six *ACS* genes (*ACS2, 4, 5, 6, 8, 11*) in 7 days old etiolated and light grown seedlings. These results were confirmed by Tsuchisaka and Theologis (2004b), who also observed an effect of IAA on the expression of *ACS7*. The effect of IAA on different *ACS* genes differed between cell types. For example, IAA induced expression of *ACS8* and *ACS11* in all cell layers of the cell expansion zone of the root, while it activated *ACS5* expression only in the endodermis. Dugardeyn et al. (2008) revealed that, with the exception of *ACS4*, the same genes are upregulated by gibberellins (GAs). Nemhauser et al. (2006) showed that *ACS6* is up-regulated by ABA and brassinolide (BL), and that *ACS10* is down-regulated by IAA, gibberellic acid (GA), and ABA, as well as by methyl jasmonate (MeJA). Finally, Wang et al. (2005) showed that *ACS4* and *ACS5* are also up-regulated by ABA, and that *ACS7* is upregulated by GA and ABA.

**FIGURE 3:**
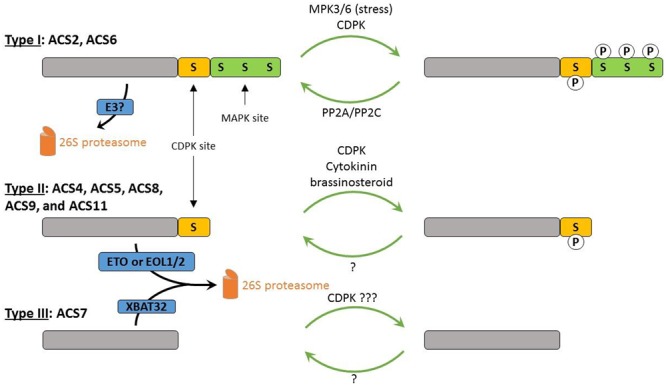
**Model for the post-translational regulation of the *Arabidopsis* ACSs.** Type I ACS proteins contain one CDPK and three MAPK phosphorylation sites. They are phosphorylated by MPK3/6 in response to external stress signals and by CDPKs, leading to stabilization and hence, enhanced ethylene production. Dephosphorylation is controlled by PP2A and PP2C. Type II ACS proteins contain one CDPK phosphorylation site and are assumed to also be more stable in their phosphorylated state. These proteins are ubiquitinated by ETO1 and EOL1/2 ubiquitin ligases. Cytokinin (or brassinosteroid) treatment has been suggested to block the ETO or EOL1/2 mediated targeting of type II ACSs. The type III ACS7 protein is potentially phosphorylated by CDPKs at its catalytic site, and is ubiquitinated by the E3 ligase XBAT32. P, phosphate group; S, serine residue.

While the transcriptional regulation of *ACS* genes is of great importance, post-translational regulation of ACS by protein degradation also plays a major role in the regulation of ethylene biosynthesis (see **Figure 3**). The C-termini, however, unimportant for enzyme activity, were found to play a crucial regulatory role in enzyme stability for proteasomal degradation. The *Arabidopsis* ACSs are classified into three groups based on the presence or absence of phosphorylation sites in these C-termini (Liang et al., 1992; Chae and Kieber, 2005). The type I ACS enzymes, ACS2 and ACS6, have a C- terminus containing phosphorylation sites for both MPKs (mitogen-activated protein kinases; Liu and Zhang, 2004) and CDPKs (calcium-dependent protein kinases; Tatsuki and Mori, 2001). The type II ACS enzymes, ACS4, ACS5, ACS8, ACS9, and ACS11, have a C-terminus containing only phosphorylation sites for CDPKs. The type III ACS enzyme ACS7 has a C-terminus without any phosphorylation sites. Phosphorylation and/or dephosphorylation of ACS proteins has a severe impact on ethylene biosynthesis. An increased phosphorylation stabilizes the ACS protein, while dephosphorylation leads to an increased proteasomal degradation (Spanu et al., 1994; Liu and Zhang, 2004; Skottke et al., 2011; Xu and Zhang, 2014).

1-aminocyclopropane-1-carboxylic acid synthase stability is controlled by two types of kinases, Ca^2+^-dependent protein kinase (CDPK) and mitogen-activated protein kinases. Types I and II ACS proteins are phosphorylated by CDPKs, as in both groups CDPK phosphorylation motifs were identified in their C-termini (Tatsuki and Mori, 2001; Sebastia et al., 2004). Type I ACS proteins are dephosphorylated by protein phosphatase 2A (PP2A) and Protein phosphatase 2C (PP2C), decreasing their protein stability (Skottke et al., 2011; Ludwikow et al., 2014). Additionally, the activity of the type I ACSs can be increased by phosphorylation by the MAP kinases MPK3 and MPK6, which have been shown to be pathogen/stress-activated (Liu and Zhang, 2004; Joo et al., 2008; Han et al., 2010). Although there are no CDPK phosphorylation sites found in the C-terminus of the type III ACS7 protein, it was recently shown that ACS7 can be phosphorylated *in vitro* in its catalytic domain (Huang et al., 2013). The E3 ubiquitin ligase targeting type-I ACSs for proteasomal degradation has not been identified yet. The degradation of the type-II ACS enzymes is mediated by ETHYLENE-OVERPRODUCER1 (ETO1) and ETO1-like 1/2 (EOL1/2), substrate-specific adaptor proteins of a Cullin4-based E3 ubiquitin ligase complex (Chae et al., 2003; Wang et al., 2004; Christians et al., 2009). The current model is that the phosphorylation of the type II proteins blocks the ability of the ETO1 and EOL1/2 proteins to bind, inhibiting the degradation of these ACSs. The degradation of the type-III ACS7 enzyme and the type-II ACS4 enzyme is mediated by XB3 ORTHOLOG 2 IN ARABIDOPSIS THALIANA (XBAT32), a RING-type E3 ligase (Prasad et al., 2010; Lyzenga et al., 2012).

Cytokinin is one of the hormones capable of regulating ethylene biosynthesis, as reflected by the elevated ethylene biosynthesis after treatment of etiolated and light grown seedlings with cytokinin (Vogel et al., 1998a,b; Woeste et al., 1999). Cytokinin does not elevate *ACS* transcript levels, but decreases the rapid degradation of ACS4 and ACS5 (Vogel et al., 1998b; Woeste et al., 1999; Chae et al., 2003). Brassinosteroids elevate ethylene biosynthesis in a similar fashion by increasing the stability of ACS5 and ACS9, and its effect is additive with that of cytokinin (Hansen et al., 2009). The stabilization of these ACSs by cytokinins and brassinosteroids includes the inhibition of the C-terminus-dependent targeting by ETO1 or EOL1/2; however, a C-terminus-independent regulation was also suggested. Hence, ACSs are regulated by other plant hormones through regulatory signals that can act together to continuously adjust ethylene biosynthesis in the different plant tissues and in response to signals from the environment.

### Regulation of the ACC Pool: Ethylene Production by ACO

While the conversion of SAM into ACC by ACS is known to be the major rate limiting step in ethylene biosynthesis, the enzyme converting ACC into ethylene (ACO) can become rate limiting under conditions of high ethylene production such as fruit ripening (Barry et al., 1996; Nakatsuka et al., 1997; Van de Poel et al., 2012; Rudus et al., 2013). In *Arabidopsis*, ACO is encoded by a small gene family of five members (*ACO1, ACO2, ACO3, EFE*, and *ACO5*). Dugardeyn et al. (2008) investigated the expression patterns of the *Arabidopsis ACO* genes using Geneatlas and AREXdb. On an organ-specific basis, *ACO1* is present at low levels in all plant organs, while *ACO2* is highly expressed allover, and *EFE* shows a strong expression in leaves. With respect to cell type- specific expression, all three genes are strongly expressed in the columella, while *ACO1* is also strongly represented in the lateral root cap, and the expression of *ACO2* is the highest in the vascular tissue of the fast elongation and differentiation zone. Though appearing in almost all vegetative and reproductive tissues, there are differences in accumulation of the members of the *ACO* gene family during different developmental and physiological processes (Barry et al., 1996; Rudus et al., 2013). In contrast to *ACS*, much less is known about the transcriptional and post-translational regulation of *ACO* genes. Similar to the *ACS*s, *ACO* expression can also be regulated by plant hormones, as reported for salicylic acid, auxins, ABA, and gibberellins (Leslie and Romani, 1988; Huang et al., 1993; Ogawa et al., 2003; Zhang et al., 2009).

### Regulation of the ACC Pool: Conjugation and Deamination

**FIGURE 4:**
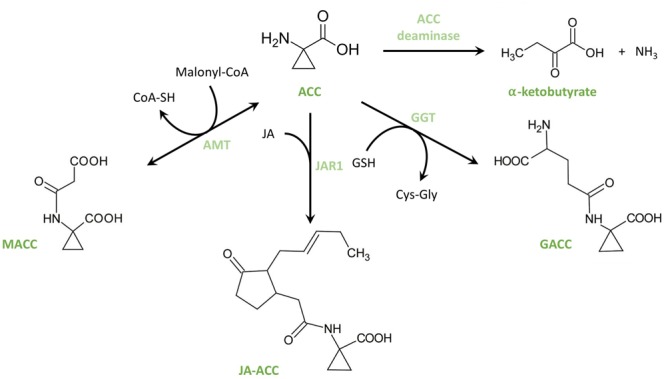
**Structural overview of ACC conjugation and deamination.** From ACC, three known conjugates can be formed. 1-malonyl-ACC (MACC) is formed by ACC-*N*-malonyl transferase (AMT), a reaction that requires malonyl-CoA. Jasmonyl-ACC (JA-ACC) is formed by jasmonic acid resistance 1 (JAR1). γ-glutamyl-ACC (GACC) is formed by γ-glutamyl-transpeptidase (GGT), a reaction that requires glutathione (GSH). The deamination of ACC by ACC deaminase yields α-ketobutyrate and ammonium.

Additional to the regulation of ethylene biosynthesis through ACS and ACO, ethylene biosynthesis is also regulated by the capability of forming ACC derivates (see **Figure 4**). Three types of conjugates have been identified so far; however, knowledge on the importance and function of these conjugates remains poor. The first ACC conjugate, *N*-malonyl-ACC (MACC), was isolated and identified independently by two research teams (Amrhein et al., 1981; Hoffman et al., 1982; Peiser and Yang, 1998). The conjugation of ACC into MACC is catalyzed by the enzyme ACC-*N*-malonyl transferase (AMT), a reaction requiring malonyl-coenzyme-A (Martin et al., 1995; Peiser and Yang, 1998). MACC can be translocated between the cytosol and the vacuole by ATP-dependent tonoplast carriers (Bouzayen et al., 1988, 1989; Tophof et al., 1989) suggesting that MACC formation and storage in the vacuoles might be important to control the pool of available ACC. This hypothesis was further strengthened by the observation from Liu et al. (1985) and Martin et al. (1995) that the production of MACC by AMT can be induced by exogenous ethylene treatments during the ripening of preclimacteric tomato (Liu et al., 1985; Martin et al., 1995). Furthermore, Jiao et al. (1986) and Hanley et al. (1989) demonstrated that MACC can be reconverted into ACC. A second conjugate that can be formed from ACC is γ-glutamyl-ACC (GACC; Martin et al., 1995). The formation of GACC is catalyzed by the enzyme γ-glutamyl transpeptidase (GGT), a reaction requiring glutathione (GSH; Martin et al., 1995; Peiser and Yang, 1998). A third ACC-conjugate is jasmonyl-ACC (JA-ACC), which is formed through the activity of the enzyme jasmonic acid resistance 1 (JAR1; Staswick and Tiryaki, 2004). This linkage between the precursors of ethylene and the active form of JA, isoleucinoyl-JA, might be a mechanism to control both hormones.

The pool of available ACC can also be reduced by the irreversible deamination of ACC (See **Figure 4**). Importantly, this reaction is not only plant-borne but also carried out by certain plant growth-promoting rhizobacteria (PGPR). Honma and Shimomura (1978) identified an ACC deaminase (ACD) in *Pseudomonas* sp. strain ACP. Plants are capable of releasing ACC into the rhizosphere to attract these PGPR which use ACC as a carbon and nitrogen source (Glick et al., 1998; Penrose and Glick, 2001; Penrose et al., 2001). As a consequence, the available ACC pool, and thus also plant ethylene production; is reduced; hence, plant growth is promoted through the plant–bacterium interaction (Glick et al., 2007a,b). In a similar manner, the reduction of the ACC pool by PGPR helps the plant to cope with stresses such as flooding (Grichko and Glick, 2001a), pathogen attack (Robison et al., 2001), or salinity (Ali et al., 2014). McDonnell et al. (2009) identified the first plant-encoded ACC deaminase and demonstrated its importance in regulating the ethylene balance.

## Acc Transport: Acc As A Water Soluble, Mobile Signal

### Long and Short Distance ACC Transport

For many hormones, the site of synthesis does not always coincide with the site of action. The same has been observed for ethylene. Because ethylene is a gaseous molecule, it diffuses rapidly through the plant tissues inducing mainly local responses, with the exception of aerenchyma through which long distance transport can be conducted, as observed in conditions of waterlogging. In contrast, long distance ethylene signaling between different plant tissues mostly occurs by the transport of ACC.

Because of its role as a stress hormone, ethylene signaling in response to biotic and abiotic stresses has been investigated thoroughly, often including a role for ACC transport. Root to shoot transport of an ethylene signal in response to waterlogging or submergence is a prime example of the importance of ACC transport and has been studies multiple times (Bradford and Yang, 1980; Metraux and Kende, 1983; Zarembinski and Theologis, 1993; Else et al., 1995; Vartapetian and Jackson, 1997; Else and Jackson, 1998; Grichko and Glick, 2001b; Almeida et al., 2003; Vriezen et al., 2003; Jackson, 2008; Dawood et al., 2016).

When plants are waterlogged or submerged, the available oxygen levels in roots drop rapidly, triggering a myriad of effects on plant metabolism. First of all, it induces a transcriptional cascade targeting genes involved in anaerobic metabolism and survival (Gibbs et al., 2011). Secondly, it induces the expression of several *ACS*s in the roots (Olson et al., 1993, 1995; Zarembinski and Theologis, 1993; Van Der Straeten et al., 1997; Shiu et al., 1998; Rieu et al., 2005). At the same time the conversion of ACC into ethylene is suppressed, because ACO requires oxygen. After root to shoot transport, ACC promotes the expression of *ACO* genes, stabilizes ACO mRNA, and increases the activity of ACOs already present, resulting in an increased ethylene production in the shoots (English et al., 1995). Differential *ACS* and *ACO* expression patterns in response flooding or hypoxia have been observed in *Arabidopsis* (Peng et al., 2005) and maize (Geisler-Lee et al., 2010). Furthermore, waterlogging and submergence leads to an overall accumulation of MACC and GACC in both roots and shoots (Amrhein et al., 1981).

The importance of ACC transport in response to waterlogging or submergence can be illustrated by research conducted in tomato (Bradford and Yang, 1980; Amrhein et al., 1981; Else et al., 1995; English et al., 1995; Else and Jackson, 1998) and in rice plants (Metraux and Kende, 1983; Zarembinski and Theologis, 1993; Kende et al., 1998; Almeida et al., 2003; Vriezen et al., 2003). Bradford and Yang (1980) showed that ACC is synthesized in tomato plant roots and then transported from hypoxic roots through the xylem to the shoots, where it is rapidly converted to ethylene inducing leaf epinasty. Furthermore, they observed that drainage results in a simultaneous decrease in ACC flux and ethylene production, and that the petioles are dependent upon the ACC flux for the high rates of ethylene synthesis. Besides epinasty, some of the other responses of plants to waterlogging and submergence, such as reduced root permeability, closure of stomata, development of aerenchyma and adventitious roots, and premature fruit drop might be directly or indirectly related to the enhancement of ACC or derivatives, besides ethylene (Vartapetian and Jackson, 1997; Grichko and Glick, 2001b; Sasidharan and Voesenek, 2015). Amrhein et al. (1982) observed that ACC can also be transported through the phloem. Based on these findings, Morris and Larcombe (1995) assessed phloem transport by foliar application of radioactive ACC to the leaves of 21 day-old cotton plants. Total radioactivity was measured over time in the stem, the hypocotyl and the apex and the patterns were consistent with phloem-mediated transport of the radioactive ACC. The authors suggested that part of the ACC which accumulates in leaves after transport through the xylem from stressed roots (Bradford and Yang, 1980) may be re-exported to other organs through the phloem. Despite the observed overall increase in the formation of the ACC conjugates MACC and GACC (Amrhein et al., 1981), leaves are apparently not capable of exporting MACC to the phloem (Morris and Larcombe, 1995). This could be a consequence of translocation of MACC to the vacuoles of leaf cells (Bouzayen et al., 1988; Tophof et al., 1989).

1-aminocyclopropane-1-carboxylic acid transport is not only important during stress conditions. Several studies evaluated the gene expression patterns of *ACS* and/or *ACO* during different developmental processes and in different organs or cell types. Woltering and associates investigated the transport of ACC in *Cymbidium* orchid flowers for the coordination of senescence (Woltering, 1990). They revealed that both endogenous and exogenously applied ACC is rapidly transported from the site of production/application to other flower parts. Jones and associates analyzed ethylene production and *ACS* and *ACO* expression patterns in different floral organs of carnation flower (*Dianthus caryophyllus L.*) in response to pollination. Ethylene production was observed in the styles, petals, and ovaries. High *ACS* expression levels were found in petals and styles, while *ACO* expression was highest in the ovaries (Jones and Woodson, 1997, 1999). In *Arabidopsis*, Dugardeyn et al. (2008) observed little *ACO* expression in the root meristematic zone, while several *ACS* genes showed high expression in this zone (*ACS3, 4, 10*, and *12*). The epidermis and endodermis of the elongation/differentiation zone also showed little *ACO* expression, while different *ACS* genes were induced. Gallie et al. (2009) investigated the cell-type specific gene expression patterns of maize root cells and found that *ACS* was only expressed in the root cortex, while *ACO* was mainly expressed in the protophloem sieve elements, suggesting ACC transport between these cell types. In all of the above-mentioned studies, the spatial differentiality in *ACS* and *ACO* expression within a given organ suggests that ACC is transported from the site of synthesis to the site of conversion to ethylene.

Shortly after the identification of ACC, the occurrence of intercellular ACC transport over short distances and intracellular compartmentalization of ACC in the vacuole was proven. Lurssen (1981) investigated ACC uptake in soybean leaf disks by measuring ethylene production after incubation with ACC. Pretreatment with ACC, followed by treatment with non-polar amino acids resulted in a reduced ethylene production, suggesting that ACC is transported by an amino acid transport system. Saftner and Baker (1987) observed the uptake of radiolabeled ACC and α-amino-iso-butyric acid (αAIB, structural analog of ACC) in tomato pericarp slices. Finally, both Tophof et al. (1989) and Saftner and Martin (1993) demonstrated the transport of ACC across the tonoplast in mesophyll cells in three different plant species (barley, wheat, and maize).

### Identification of a First ACC Transporter, LHT1

**FIGURE 5:**
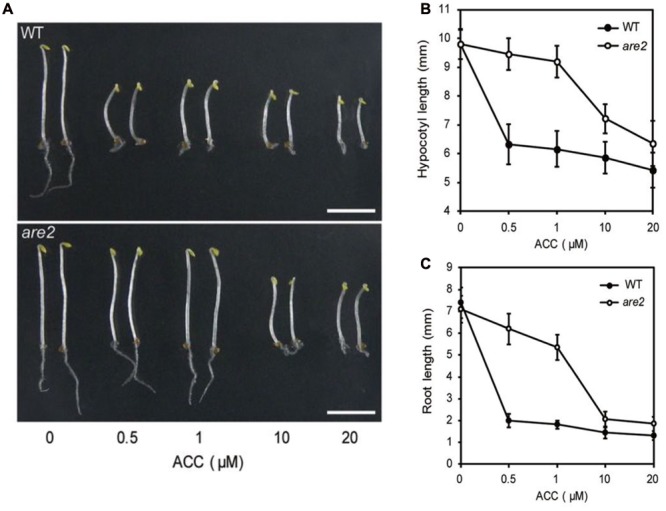
**Dose-dependent response of *Arabidopsis thaliana* seedlings to exogenously applied ACC suggesting possible involvement of ARE2/LHT1 in ACC transport.** Wild type and *are2* mutants were grown in the darkness for 4 days on Murashige and Skoog (MS) medium containing various ACC concentrations (0, 0.5, 1, 10, and 20 μM ACC). Scale bar = 5 mm. **(A)** Representative phenotypes for the wild type (WT) and *are2* mutant. **(B,C)** Hypocotyl lengths and root lengths of seedlings grown as in **(A)**. Figure reproduced from Shin et al. (2015).

In a recent study, a first plant ACC transporter was found. Shin et al. (2015) identified an *Arabidopsis thaliana* mutant, *are2* (*ACC-resistant2)*, showing a dose-dependent resistance to exogenously applied ACC. In the light, the wild type shows an elongated hypocotyl and shortened root growth in the presence of 1 μM exogenous ACC (Smalle et al., 1997), while the *are2* mutant exhibited a reversed phenotype, with a shorter hypocotyl and more elongated roots. When grown in darkness in the presence of 1 μM ACC the hypocotyls and the roots of the *are2* mutant were not significantly inhibited as compared to the wild type. However, at elevated concentrations of ACC (10 μM or above) a triple response was observed, indicating sensitivity of the *are2* mutant (**Figure 5**). Interestingly, the mutant displayed normal sensitivity to ethylene, and the synthesis of ethylene was not impaired, neither did the insensitivity to ACC affect sensitivity of the seedlings to other plant hormones. The *are2* mutant corresponded to a null allele of *LHT1* (*LYSINE HISTIDINE TRANSPORTER 1*), a plasma membrane (PM)-localized amino acid transporter. LHT was first identified by Chen and Bush (1997), and further characterized by Hirner et al. (2006). To confirm that *are2* and *LHT1* are allelic, Shin et al. (2015) performed an allelism test with the *lht1-5* mutant. The double mutant and the single mutants showed the same ACC resistance and early leaf senescence phenotypes. An uptake experiment using radiolabeled ACC and protoplasts from wild type and *are2/lht1-5* mutant leaf mesophyll cells, showed a reduction of 40% in ACC uptake in both mutants, supporting a function of LHT1 in ACC uptake transport. In *Arabidopsis*, LHT1 is part of a family of amino acid transporters with 10 members (AtLHT1-10), all of which are localized to the plasma membrane and transport a broad spectrum of amino acids from the cell wall space inward (Hirner et al., 2006). The *LHT* genes show distinct and overlapping expression patterns, indicating that LHT1 homologs may function in ACC uptake under certain developmental or environmental conditions (Foster et al., 2008). Based on unpublished results, Shin et al. (2015) suggest that at least one *LHT1* homolog could complement the *lht1* mutant.

## Functions Of Acc As An Ethylene-Independent Signal

The secretion of ACC into the rhizosphere to attract and interact with PGPR, as discussed in the previous section, is an example of an ethylene-independent function for ACC. Recent studies present evidence that ACC, or one of its derivates, might also have other signaling roles independent from ethylene (Yoon and Kieber, 2013).

First, the leucine-rich repeat receptor-like protein kinases (LRR-RLKs) FEI1 and FEI2 appear to define an ACC-mediated signaling pathway that regulates cell wall function and anisotropic cell expansion (Xu et al., 2008). The kinase domains of both FEI isoforms interact with the type II ACS proteins ACS5 and ACS9 (Chae and Kieber, 2005) without affecting their catalytic activity (Xu et al., 2008). They found that mutation of both *FEI* genes perturbs the biosynthesis of cell wall polymers. Phenotypic analysis of light-grown seedlings showed that although single *fei1* and *fei2* mutants are indistinguishable from the wild type, the *fei1fei2* double mutant has short, radially swollen roots, and a significant, but less pronounced swelling of the hypocotyl, indicating a defective anisotropic expansion. This expansion defect was suppressed by inhibition of ACS with aminooxyacetic acid (AOA), but not by disruption of the ethylene response pathway with 1-methylcyclopropene (1-MCP) or silver thiosulfate, neither by mutation of ETR1 (an ethylene receptor) or EIN2 (a central regulator of ethylene signaling). All together, these results suggest that the FEIs do not act through ethylene, but rather through ACC. Finally, Xu et al. (2008) also proposed that FEI proteins might act as a scaffold to localize ACS or assemble ACS in a protein complex.

Further evidence for a signaling role for ACC came from a detailed study of multiple loss of function of *ACS* genes (Tsuchisaka et al., 2009). Strong alleles of octuple mutants displayed embryo lethality as well as reduced branching, none of which are observed in ethylene signaling mutants (Alonso et al., 1999; Tsuchisaka et al., 2009; Tsang et al., 2011). These phenotypic differences between ethylene biosynthesis and signaling mutants once again suggest that ACC or one of its derivates might act as a signal independent from ethylene.

A third study relates ACC signaling to primary root elongation in response to pathogen-associated molecular patterns (PAMPs). Tsang et al. (2011) studied the acute response of plants to perturbation of cell wall integrity using inhibitors of cellulose biosynthesis. Application of AOA, aminoethoxyvinylglycine (AVG), or 2-anilino-7-(4-methoxyphenyl)-7,8-dihidro-5(6H)-quinazolinone (7303; ACS inhibitors) or AIB (ACO inhibitor) could revert the suppression of root cell expansion caused by isoxaben, a blocker of cellulose synthesis. Similar experiments using silver ions or norbornadiene, interfering with ethylene perception, did not result in a significant effect on elongation. Additionally, they present that not only isoxaben, but also ACC, has an effect on root elongation both in the ethylene insensitive *ein3eil1* mutant and the wild type. These results suggest that ACC has a short-term effect on root elongation that is partially independent of its conversion to ethylene or ethylene signaling, and that it is responsible for the rapid reduction of root elongation triggered by PAMPs.

Considering the above-mentioned observations, a signaling role for ACC independent from ethylene is highly likely. Whether it is ACC itself serving as a signal, or one of its derivatives, is still a matter of debate and needs further investigation.

## Considerations for Future Research

### Co-regulation of Ethylene Biosynthesis Genes and ACC Transporters?

The identification of LHT1 as an ACC transporter is a cornerstone in unraveling the mechanism and regulation of ACC transport in plants. Giving the complexity of ethylene biosynthesis and signaling, and the evidence that ACC can be transported both over long distances between plant organs and over short distances within or between cells, one can assume that ACC transport involves several proteins, presumably even different transport systems. It is thus quite possible that many more ACC transporters are yet to be identified. Performing uptake experiments of the *are2* mutant using radiolabeled ACC, Shin et al. (2015) observed an incomplete reduction of ACC uptake, supporting the presence of additional ACC transporters.

**FIGURE 6:**
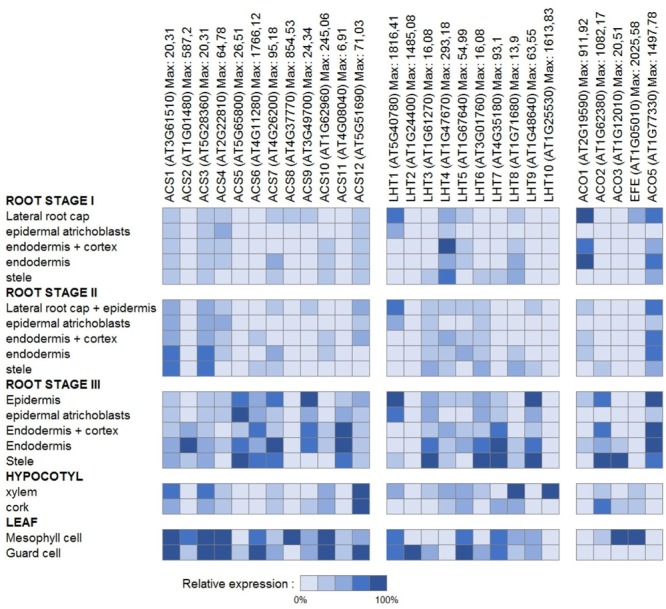
**Gene expression of ethylene biosynthesis genes and genes of the LHT family of amino acid transporters.** Cell type-specific relative expression patterns are presented for the root, the hypocotyl, and the leaf. The root stages indicate the developmental stages as described by (Birnbaum et al., 2003) for 6 days old seedlings. Stage I: where the root tip reached its full diameter (about 0.15 mm from the root tip); stage II: where cells begin longitudinal expansion (about 0.30 mm from the root tip); stage III: where root hairs are fully elongated (0.45 to 2 mm from the root tip). For each gene, the cell type-specific expression levels presented were computed relative to the maximal absolute expression, which can be found behind each gene name.

**FIGURE 7:**
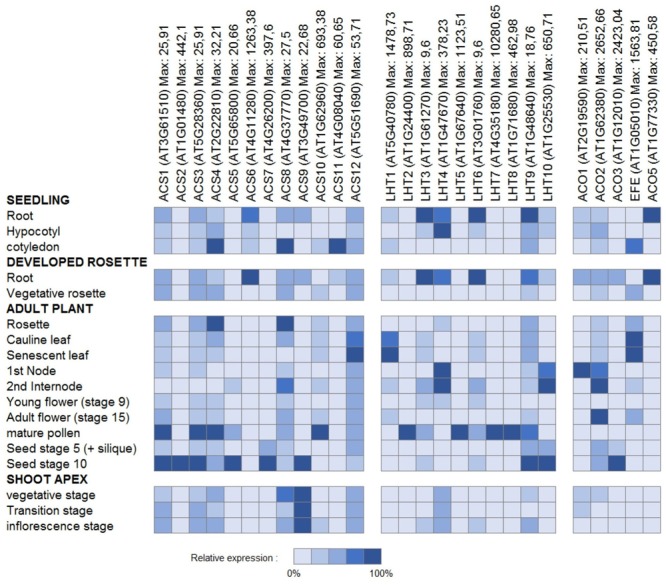
**Gene expression of ethylene biosynthesis genes and genes of the LHT family of amino acid transporters.** Relative expression patterns are presented for given tissues in different developmental stages. For each gene, the relative expression levels presented were computed relative to the maximal absolute expression, which can be found behind each gene name.

Aiming at a more profound understanding of ACC transport, it is tempting to look at the organ and cell-type specific gene expression patterns of the ethylene biosynthesis genes *ACS* and *ACO* as a main determinant of ACC accumulation. In tissues with high *ACS* expression, where, however, little or no *ACO* expression is found, ACC export might be important. In contrast, in tissues with low *ACS* expression, though high *ACO* expression, ACC import might be important. For an overview of the detailed expression patterns for both enzyme families, we extracted cell-type specific and developmental stage specific absolute expression values from the Toronto Arabidopsis eFP browser (BAR). The relative expression percentages, as presented in **Figures 6** and **7**, were calculated for each gene, relative to the expression value of the cell-type with the highest absolute value.

**Figure 6** presents an overview of the cell type-specific expression levels in the roots, the hypocotyl and the leaves. Little *ACO* expression is found in the root epidermal atrichoblasts, hypocotyl xylem, and leaf guard cells, while some *ACS*s are highly expressed in these cell-types. This suggests that the ACC produced is most likely transported to other tissues (with higher *ACO* expression) where it is converted into ethylene. It has to be acknowledged, however, that these expression patterns do not give much information about long distance or intracellular transport of ACC. **Figure 7** presents an overview of the relative expression levels for certain tissues of different developmental stages. These expression patterns show in which developmental tissues ethylene production is important, but they also indicate between which tissues the translocation of ACC might occur. For example, in young flowers the *ACO*s are barely expressed, while in mature flowers increased expression levels are observed for both *ACO2* and *EFE*. This means that ethylene production is most likely higher in mature flowers. In addition, there is little difference in the expression levels of the *ACS*s between these two developmental stages. This might suggest that the increased ethylene production in mature flowers is dependent on the supply of ACC from other tissues. In pollen, high levels of *ACS* expression can be found, while there is almost no ACO expression. This discrepancy corresponds with the observation that high levels of ACC are present in mature pollen, and that this pollen-ACC may be an important mediator of the early response of flowers to pollination (Whitehead et al., 1983; Reid et al., 1984). This was confirmed by (Reid et al., 1984), with the observation that application of ACC to the stigmas of carnation flowers (*Dianthus caryophyllus*), causes an initial increase in gynoecium and petal ethylene production similar to that reported for pollinated flowers, and that application of [2-^14^C]ACC to the stigmas resulted in radioactive ethylene production both by the gynoecia and the petals. This report provides additional evidence that during pollination (and post-pollination) ACC is exported from the pollen, and transported toward different flower tissues, where it is converted into ethylene.

The *ACS* and *ACO* gene expression patterns as described above can be compared with those of known transporters to find novel ACC transporters. There are two major paths that can be followed here. On the one hand, one can look at members of general transporter families such as the family of ATP binding cassette (ABC) transporters, of which some members are important for the transport of IAA, ABA, CK, and SL (Xu et al., 2014; Borghi et al., 2015). One the other hand one can look at known amino acid transporters. The *Arabidopsis* genome contains 60 or more predicted amino acid transporters, belonging to at least two large families.

One of these amino acid transporter families is the LHT family. Previous research already showed that *LHT1* is expressed in roots and mesophyll cells, and that *LHT2* expression is localized to the tapetum of anthers (Chen and Bush, 1997; Lee and Tegeder, 2004). **Figures 6** and **7** present an overview of the cell type-specific and development specific expression patterns for the *Arabidopsis LHT*s. *LHT1* expression is high in epidermal atrichoblasts and guard cells, two cell types in which ACC transport was suggested to be important (see above). This is in part a confirmation that in these tissues ACC transport could be supported by LHT1 action. *LHT2, LHT5*, and *LHT7* also show high expression in guard cells, while *LHT8* and *LHT10* show high expression in the hypocotyl xylem. In the pollen, where ACC export might be essential for the induction of pollination, *LHT2, 5, 8*, and *9* are highly expressed. These results reveal that certain other members of the LHT family might also be linked with ACC transport. However, as they are also amino acid transporters, these expression patterns might not necessarily mean that this is the case.

### ACC Transport by Other Amino Acid Transporters?

Because ACC is a non-protein amino acid, it seems evident that ACC transporter(s) would have the characteristics of an amino acid transporter, or be transported by a protein that serves in transport of other amino acids as well. In an assay measuring ethylene upon incubation of leaf disks with ACC, Lurssen (1981) tested competition of other amino acids with ACC. Uptake of ACC was inhibited by L-amino acids with non-polar side chains such as L-methionine, L-tyrosine, and L-phenylalanine, but not by polar amino acids. These were the first indications that ACC might be transported by an amino acid transporter. Saftner and Baker (1987) investigated ACC transport in tomato pericarp slices, and observed that the uptake was inhibited by neutral amino acids, with the highest inhibition from methionine. Given that ACC uptake can be inhibited by the competition of other amino acids, it might be of interest to investigate whether other known amino acid transporters are also capable of transporting ACC, for instance by a systematic analysis using heterologous expression in yeast or in protoplasts. ACC transport could be assayed using radiolabeled ACC or by measuring ethylene production.

Besides comparing gene expression patterns of amino acid transporters, as was suggested in the previous section, another approach is to look at the amino acid substrates of the different transporters. As discussed earlier, ACC transport can be suppressed by neutral and/or non-polar amino acids (Lurssen, 1981; Saftner and Baker, 1987). Amino acid transporters having a high affinity for one or more of these amino acids might be considered potential candidates for ACC transport.

### Identification of Additional ACC Transporters

The studies discussed in the previous two sections assume that novel ACC transporters can be found in families of known transporters. It is, however, quite possible that some ACC transporters remain uncharacterized to date. For identification of novel transporters several approaches could be taken. Most of our current knowledge on plant transporters has been obtained by use of the yeasts *Saccharomyces cerevisiae* and *Schizosaccharomyces pombe* (Dreyer et al., 1999). Using one of these heterologous expression systems, cDNA libraries can be screened to identify novel ACC transporters. An alternative approach is a forward genetic screen using the chemical mutagen ethyl methanesulfonate (EMS). For this forward genetic screen, the phenotypic analysis might include the screening for ACC resistance (dose-dependent or not) as seen by Shin et al. (2015) for *are2/lht1-5*.

### Long-Distance Transport of ACC Conjugates and Their Role in Ethylene Biology

Several studies explored the possibility of transport of ACC conjugates. Fuhrer and Fuhrer-Fries (1985) investigated the formation and the transport of ACC and MACC in pea plants after wounding, which normally induces an increase of ethylene biosynthesis. They concluded that MACC could be transported from the shoot to the root and that the roots act as an MACC sink. However, further investigation is needed to rule out the possibility that the MACC increase at the roots may result from *de novo* MACC production after ACC transport from the shoot to the roots. Furthermore, the MACC in the shoot could be reconverted into ACC. Finlayson et al. (1991) investigated the transport and metabolism of ACC in sunflower seedlings. This study presents evidence for the presence of MACC in xylem sap of seedlings treated with radiolabeled ACC. However, the presence of MACC in the xylem sap could not be confirmed by GC-MS analysis of untreated seedlings. These results suggest that MACC can be transported through the xylem, but that this transport might only occur during certain stress conditions. Morris and Larcombe (1995) investigated the possibility of MACC transport through the phloem in cotton. In contrast to the previously discussed studies, they found no evidence for MACC transport.

Overall it is clear that additional evidence is needed to confirm a more general nature of MACC transport, and add to our understanding of its biological function in plants. Moreover, it is also important to further investigate whether the other ACC conjugates can be transported, or whether they rather function as a storage forms of ACC to regulate ethylene production within the organ or tissue where they are formed.

## Possible Applications In Agriculture

With the rapidly increasing world population and concomitant food demand, more efforts are being made to increase crop yield and crop product quality all over the world. As discussed previously, ethylene regulates a wide variety of vegetative and developmental processes in plants, all being key processes in agricultural and horticultural context.

Several chemicals regulate these ethylene-dependent characteristics either through the inhibition of ethylene synthesis or perception [aminoethoxyvinylglycine (AVG; Byers et al., 2005; Drake et al., 2006), aminooxyacetic acid (AOA; Broun and Mayak, 1981), diazocyclopentadiene (DACP; (Blankenship and Sisler, 1992), silver thiosulphate (STS; Hansen et al., 1996; Hoyer, 1998) and 1-methylcyclopropene (1-MCP; Drake et al., 2006; Zhu et al., 2015)], or through the release of ethylene from 2-chloroethylphosphonic acid (Ethephon; Logendra et al., 2004; Drake et al., 2006). Treatment with inhibitors of ethylene biosynthesis can reduce postharvest senescence of leafy vegetables (Able et al., 2003; Lomaniec et al., 2003) and increase shelf life of cut flowers and potted plants (In et al., 2015; Silva and Finger, 2015). In climacteric fruit, preharvest treatment with inhibitors of ethylene biosynthesis can delay the initiation of ripening to increase fruit quality and reduce early fruit abscission of for example apple (Hofman et al., 2001; Drake et al., 2006), pear (Villalobos-Acuna et al., 2010), avocado (Hofman et al., 2001; Salazar-Garcia et al., 2006), mandarin (Nawaz et al., 2008), papaya (Hofman et al., 2001), and mango (Hofman et al., 2001). Conversely, postharvest treatment can reduce loss of fruit quality due to over-ripening (Drake et al., 2006; Zhu et al., 2015). Preharvest treatment with inducers of ethylene synthesis reduces the harvest window and increases crop uniformity (Logendra et al., 2004; Ampa et al., 2016). However, because these inducers of ethylene synthesis are not tissue specific, they will promote senescence and abscission in other plant organs making them unsuitable for use on perennial plants which produce clusters of fruit sequentially over several months or years (Logendra et al., 2004).

The use of chemical blockers or inducers of ethylene biosynthesis is expensive, impractical in natural soil and their effect on human health and/or the environment is still ambiguous. The latter led to the banishment of some of these products from agricultural practices in several countries, and more may follow (Scariot et al., 2014; Tacuri et al., 2016). This means that there is a need for better alternatives. With the elucidation of the ACC transport mechanisms in plants, ethylene production could be fine-tuned with an improved specificity. The transport of ACC toward certain plant organs could be induced/blocked without affecting development of other organs. Possible applications can be envisaged for all processes that are known to be dependent on ACC transport. These include the above-mentioned conditions where chemical ethylene blockers/releasing agents are currently used, such as the regulation of the timing of fruit ripening and/or abscission, flower abscission and/or senescence, as well as leaf senescence. Furthermore, it will also be possible to synchronize harvest time in perennial crop species in which the use of chemical ethylene releasing compounds is not possible. Overall, it will allow these perennials to recuperate better and faster after each subsequent harvesting period. With the increasing interest for the use of these perennial crops, this application will have significant impact on the agricultural sector.

In addition to the above-mentioned fine-tuning of ACC transport as alternative for ethylene blocking/releasing compounds, it also has an application potential in mitigation of the effects of environmental stresses. Exploration of new options to reduce the vulnerability of our agricultural systems to these stresses becomes critical. As discussed previously, conditions such as drought, flooding, heat, and salinity have been linked with long distance root to shoot transport of ACC and the subsequent elevation of ethylene production (Morgan and Drew, 1997; Schachtman and Goodger, 2008; Sasidharan and Voesenek, 2015; Tao et al., 2015). Consequently, under these stress conditions, plant growth and developmental processes are influenced severely, leading to significant yield losses, the burden of which can only increase as a result of global change. By blocking long distance ACC transport from the root to the shoot in response to environmental stress signals, ethylene-induced negative effects on crop yield could be minimized. Appropriate constructs with tissue/cell type specific promotors could be envisaged once specific transporters are identified. However, it has to be noticed equilibrated impact on ACC transport might be needed, in order to reach the optimal balance between the adaptive response of the plant (needing ethylene as a signal) and the rescue of its normal growth (impeded by ethylene). Another path to be explored relates to the beneficiary effect of PGPR (Barea et al., 2005), a subset of which can stimulate plant growth as a result of conversion of ACC exported by the plant toward the rhizosphere. PGPR colonize the roots of monocots and dicots, and influence many aspects of plant life including nutrition, growth, morphogenesis and response to biotic and abiotic stress by direct and indirect mechanisms. Hence, the identification of specific ACC exporters in the root epidermis could open several avenues for applications in the area of biostimulants.

To tune ACC transport in order to control a specific response, an approach based on site-specific genome editing would be most promising. Of all current site-specific genome editing technologies, CRISPR/Cas9 shows the greatest potential for crop improvement (Belhaj et al., 2015). Cas9 is an RNA-guided DNA endonuclease which can be targeted to specific genomic sequences by expression of a customized guide RNA with which it is capable of forming a complex. If the double-strand breaks (DSBs) resulting from the action of Cas9 are imperfectly repaired by the plant’s endogenous non-homologous end joining (NHEJ) repair mechanism, gene function can be disrupted resulting in knock-down or knock-out lines. The technique has already successfully been applied in a variety of plant species, with efficiency rates higher than 90% in *Arabidopsis* (Feng et al., 2014) and rice (Miao et al., 2013). In addition, this tool allows multiplex editing, meaning that multiple sites can be targeted at the same time (Li et al., 2013). A second possible approach for the regulation of ACC transport is the use of tools altering the epigenetic regulation, i.e., without altering the primary DNA sequence (incl. DNA methylation, histone modification) of ACC transporters. Two examples of such epigenetics tools are RNA-directed DNA methylation (RdDM) and RNA interference (RNAi). RdDM induces transcriptional gene silencing by methylation of promoter sequences (Chinnusamy and Zhu, 2009), thus allowing epigenetic modification of gene expression in crop species (Hartung and Schiemann, 2014). In contrast to the genome editing technique CRISPR/Cas9, however, these epigenetic changes are only stable for a few generations (Hartung and Schiemann, 2014). RNAi is a commonly used technique allowing the transcriptional and post-transcriptional silencing of a gene of interest by small interfering RNAs (siRNAs) (Small, 2007). These siRNAs are capable of binding with a complementary sequence in a messenger RNA (mRNA) molecule, inducing its subsequent cleavage. In addition, siRNAs have been linked with changes in heterochromatin formation inducing transcriptional gene silencing (Djupedal and Ekwall, 2009).

## Conclusions and Perspectives

Since the discovery of ethylene as a plant hormone and ACC as its direct precursor, accumulated evidence supports a central role for ACC in many aspects of ethylene synthesis, but also beyond ethylene biology. In order to advance our understanding of ACC as a pivotal molecule in plant growth and development, a few key issues need to be addressed. First, a better understanding of the transcriptional or post-translational control of ACO is necessary. Foremost, a profound understanding of ACC transport, including identification of genes encoding different types of transporters, together with a detailed organ-specific metabolomics study of ACC and its conjugates, would help to draw a map of ACC traffic within the plant body. As for ACC itself, besides the mechanisms of its transport, its possible role as a signaling molecule is an additional challenging route with a lot of potential.

For the identification of novel ACC transporters we suggest three potential approaches. The first focusses on a gene expression study assuming that ACC transporters show high expression at sites of differential *ACS* and *ACO* expression, the second concentrates on analysis of the affinities of known amino acid transporters, while the third aims at the identification of yet uncharacterized transporters using a heterologous expression system or a forward genetic screen. With the information gained from detailed ACC transporter analyses, agricultural applications can be explored, including the regulation of ACC transport as an alternative for ethylene releasing/blocking chemicals, or as a way to alleviate the increasing negative effects of environmental stresses on crop yield. Fine tuning of ACC transport could be obtained by using novel techniques for gene modification. These include CRISPR/Cas9 genome editing technology for the site specific mutagenesis of a gene of interest and epigenetic tools altering the transcriptional and post-transcriptional regulation of a gene of interest.

## Author Contributions

All authors listed, have made substantial, direct and intellectual contribution to the work, and approved it for publication.

## Conflict of Interest Statement

The authors declare that the research was conducted in the absence of any commercial or financial relationships that could be construed as a potential conflict of interest.
